# Global increase and geographic convergence in antibiotic consumption between 2000 and 2015

**DOI:** 10.1073/pnas.1717295115

**Published:** 2018-03-26

**Authors:** Eili Y. Klein, Thomas P. Van Boeckel, Elena M. Martinez, Suraj Pant, Sumanth Gandra, Simon A. Levin, Herman Goossens, Ramanan Laxminarayan

**Affiliations:** ^a^Center for Disease Dynamics, Economics & Policy, Washington, DC 20005;; ^b^Department of Emergency Medicine, Johns Hopkins School of Medicine, Baltimore, MD 21209;; ^c^Department of Epidemiology, Johns Hopkins Bloomberg School of Public Health, Baltimore, MD 21205;; ^d^Institute of Integrative Biology, ETH Zürich, CH-8006 Zürich, Switzerland;; ^e^Department of Ecology and Evolutionary Biology, Princeton University, Princeton, NJ 08544;; ^f^Princeton Environmental Institute, Princeton University, Princeton, NJ 08544;; ^g^Beijer Institute of Ecological Economics, SE-104 05 Stockholm, Sweden;; ^h^Laboratory of Medical Microbiology, Vaccine & Infectious Diseases Institute, University of Antwerp, 2610 Antwerp, Belgium;; ^i^Department of Global Health, University of Washington, Seattle, WA 98104

**Keywords:** antimicrobial resistance, low-income countries, defined daily doses, antibiotic stewardship, antibiotics

## Abstract

Tracking antibiotic consumption patterns over time and across countries could inform policies to optimize antibiotic prescribing and minimize antibiotic resistance, such as setting and enforcing per capita consumption targets or aiding investments in alternatives to antibiotics. In this study, we analyzed the trends and drivers of antibiotic consumption from 2000 to 2015 in 76 countries and projected total global antibiotic consumption through 2030. Between 2000 and 2015, antibiotic consumption, expressed in defined daily doses (DDD), increased 65% (21.1–34.8 billion DDDs), and the antibiotic consumption rate increased 39% (11.3–15.7 DDDs per 1,000 inhabitants per day). The increase was driven by low- and middle-income countries (LMICs), where rising consumption was correlated with gross domestic product per capita (GDPPC) growth (*P* = 0.004). In high-income countries (HICs), although overall consumption increased modestly, DDDs per 1,000 inhabitants per day fell 4%, and there was no correlation with GDPPC. Of particular concern was the rapid increase in the use of last-resort compounds, both in HICs and LMICs, such as glycylcyclines, oxazolidinones, carbapenems, and polymyxins. Projections of global antibiotic consumption in 2030, assuming no policy changes, were up to 200% higher than the 42 billion DDDs estimated in 2015. Although antibiotic consumption rates in most LMICs remain lower than in HICs despite higher bacterial disease burden, consumption in LMICs is rapidly converging to rates similar to HICs. Reducing global consumption is critical for reducing the threat of antibiotic resistance, but reduction efforts must balance access limitations in LMICs and take account of local and global resistance patterns.

## Significance

Antibiotic resistance, driven by antibiotic consumption, is a growing global health threat. Our report on antibiotic use in 76 countries over 16 years provides an up-to-date comprehensive assessment of global trends in antibiotic consumption. We find that the antibiotic consumption rate in low- and middle-income countries (LMICs) has been converging to (and in some countries surpassing) levels typically observed in high-income countries. However, inequities in drug access persist, as many LMICs continue to be burdened with high rates of infectious disease-related mortality and low rates of antibiotic consumption. Our findings emphasize the need for global surveillance of antibiotic consumption to support policies to reduce antibiotic consumption and resistance while providing access to these lifesaving drugs.

Antibiotic resistance—the ability of microbes to evolve and withstand the effects of antibiotics—is a significant cause of morbidity and mortality globally (1–3). Antibiotic consumption is a primary driver of antibiotic resistance (4). The association between antibiotic consumption and resistance is well documented across spatial and temporal scales at individual hospitals (5), nursing homes (6), primary care facilities (7), and communities (8), as well as across countries (9). Many countries have adopted national action plans on antimicrobial resistance (AMR) that aim to reduce per capita antibiotic consumption. The Global Action Plan on Antimicrobial Resistance endorsed by the member states of the World Health Organization (WHO) and affirmed at the high-level meeting on antimicrobial resistance during the 71st General Assembly of the United Nations (10), recommends that all countries collect and report antibiotic consumption data (11).

Surveillance data on country-level antibiotic use are needed to (*i*) monitor national and global trends over time; (*ii*) compare antibiotic use among countries; (*iii*) provide a baseline for the evaluation of future efforts to reduce antibiotic use; (*iv*) enable epidemiological analysis of the association between antibiotic use and resistance over time (12, 13); and (*v*) support policies that aim to reduce antibiotic resistance. Given the urgency of the threat posed by rising AMR levels (2), and in the absence of global, publicly funded, harmonized surveillance data on antibiotic use, alternative sources of data on antibiotic use must be used to track antibiotic consumption patterns across countries. Here, we use pharmaceutical sales data to document global trends in antibiotic consumption.

There have been few attempts to assess antibiotic consumption globally or in multiple countries (14–17), none of which has reported data later than 2010. The largest prior study reported that antibiotic consumption increased 36% between 2000 and 2010 in 71 countries (15). However, the results from this study cannot be directly compared with other studies (14, 16) because the data were not reported as defined daily doses (DDDs), the most commonly used metric to measure antibiotic consumption. In this study, we report on antibiotic consumption in DDDs for an expanded number of countries (*n* = 76) and over a longer time period (2000–2015). In addition, we assess differences in consumption between high-income countries (HICs) and low- and middle-income countries (LMICs), identify drivers of antibiotic use in each income group from a set of socioeconomic and medical indicators, and project future growth in global antibiotic consumption.

## Methods

We estimated global antibiotic consumption using the IQVIA MIDAS database. IQVIA uses national sample surveys of antibiotic sales to develop estimates of the total volume of sales of each antibiotic molecule (or combination of molecules). For each country, antibiotic consumption was reported by month or quarter and broken down between the retail and hospital sectors. We obtained data for 76 countries from 2000 through 2015. Central America (Costa Rica, El Salvador, Guatemala, Honduras, Nicaragua, and Panama) and French West Africa (Benin, Burkina Faso, Cameroon, Chad, Côte d’Ivoire, Republic of Congo, Guinea, Mali, Niger, Senegal, and Togo) were included as two individual groups of countries with aggregated sales for these regions. Sixty-six countries had data available for every year between 2000 and 2015, while data on the remaining countries covered partial time periods (*SI Appendix*, Table S1). In countries where both hospital and retail data were reported for some but not all years (2000–2015), consumption in the missing sector was estimated by interpolation, using the ratio of antibiotic consumption in the hospital and retail sectors for the years for which data had been reported.

Data on antibiotic sales in standard units (SUs) and kilograms were purchased under license from IQVIA. An SU is an IQVIA designation that represents a single-dose unit such as a pill, capsule, or equal amount of liquid. Sales expressed in kilograms were converted into DDDs using the Anatomical Therapeutic Chemical Classification System (ATC/DDD, 2016) developed by the WHO Collaborating Centre for Drug Statistics Methodology. For molecules not included in the ATC/DDD index, DDD values were estimated from other sources or as the average of DDD unit values by class (*SI Appendix*, *SI Methods*). Data for SUs were available for all years, whereas kilogram data were available only for the period 2005–2015. The ratio of SUs to kilograms for 2005–2015 was used to estimate kilograms and DDDs for 2000–2004. A country’s annual antibiotic consumption rate in DDDs per 1,000 inhabitants per day was calculated using population estimates from the World Bank DataBank. Consumption rates were subsequently compared between groups of countries based on their World Bank income classification in 2007.

Fixed-effects panel regression analysis was used to quantify the association between economic and health indicators and the antibiotic consumption rate. The explanatory variables included per capita gross domestic product (GDP; purchasing power parity); imports of goods and services as a percentage of GDP (as a measure of trade); measles vaccination coverage in children between the ages of 12 and 23 mo [lack of pneumococcal conjugate vaccination (PCV) coverage information limited our ability to include PCV coverage as a variable]; physician density per 1,000 population; and percentage of the population living in urban areas (as a measure of health system access). All explanatory variables were pooled by country/year. Regression models analyzed data for HICs and LMICs separately. Health and economic factors were obtained from the World Development Indicators in the World Bank DataBank (18). Serial correlation was assessed using the Wooldridge test (19). Errors were clustered by country to account for high serial correlation. STATA version 14.1 was used for all statistical analyses.

Global antibiotic consumption was calculated by extrapolating use for countries not included in the IQVIA database. Extrapolations were based on the average per capita antibiotic use for countries from the same income group. We then projected global antibiotic use until 2030, assuming constant per capita use rates for all countries at current levels, with total use increasing only through population growth. In addition to this baseline projection, we modeled two other scenarios: (*i*) no policy changes, where countries’ antibiotic consumption rates for 2016 through 2030 were assumed to continue to change based on their compounded growth rate between 2010 and 2015 and (*ii*) a target policy in which countries were assumed to converge to the 2015 global median antibiotic consumption rate by 2020 (*SI Appendix*, *SI Methods*). Population projections were retrieved from the World Bank DataBank (18) except for Taiwan, for which data were obtained from the Taiwan National Development Council.

## Results

Global antibiotic consumption increased by 65% between 2000 and 2015, from 21.1 to 34.8 billion DDDs, while the antibiotic consumption rate increased 39% from 11.3 to 15.7 DDDs per 1,000 inhabitants per day over the study period. The mean antibiotic consumption rate across countries increased 28% from 16.4 (SD 9.9) DDDs per 1,000 inhabitants per day to 20.9 (SD 9.8), and the median antibiotic consumption rate increased 25% from 15.5 to 19.5 DDDs per 1,000 inhabitants per day.

**Fig. 1.**
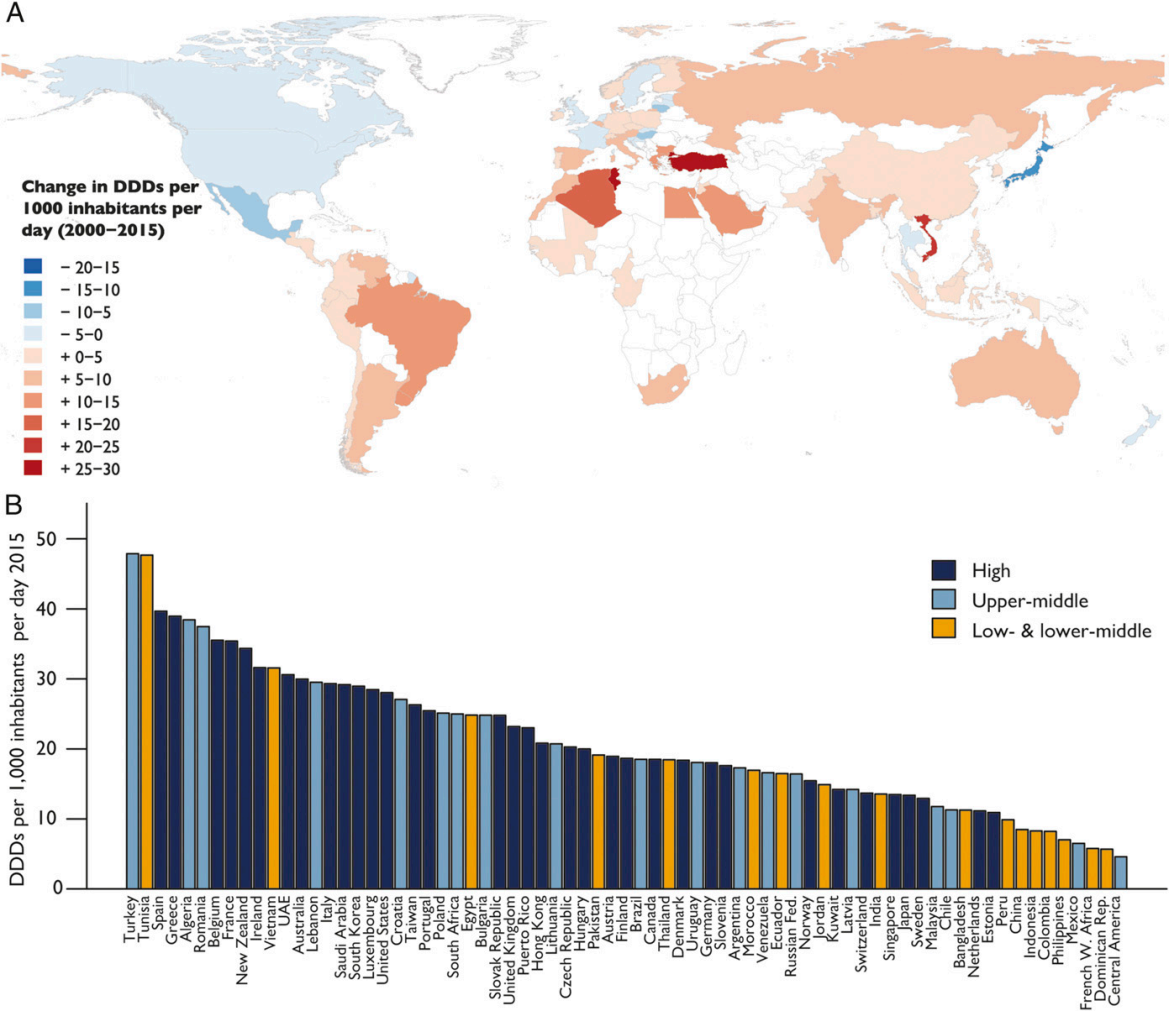
Global antibiotic consumption by country: 2000–2015. (*A*) Change in the national antibiotic consumption rate between 2000 and 2015 in DDDs per 1,000 inhabitants per day. For Vietnam, Bangladesh, The Netherlands, and Croatia, change was calculated from 2005, and for Algeria from 2002 as data before those years for those countries were not available. (*B*) Antibiotic consumption rate by country for 2015 in DDDs per 1,000 inhabitants per day. Data source: IQVIA MIDAS, 2000–2015, IQVIA Inc. All rights reserved (https://www.iqvia.com/solutions/commercialization/geographies/midas).

**Fig. 2.**
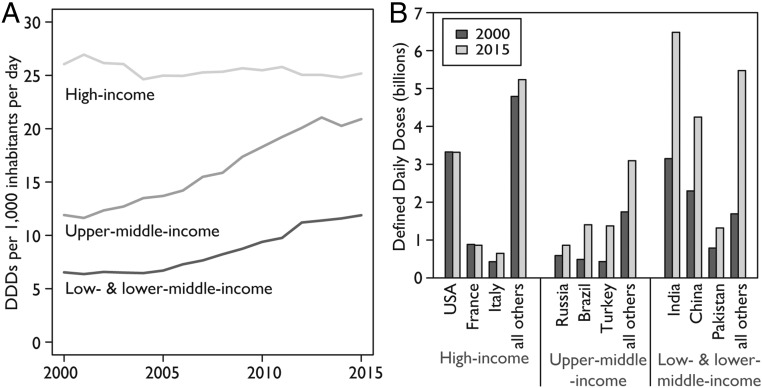
Global antibiotic consumption by country income classification: 2000–2015. (*A*) Graph showing how the antibiotic consumption rate in DDDs per 1,000 inhabitants per day has rapidly increased for LMICs, while remaining nearly constant for HICs. However, as shown in *B*, the larger population sizes in many LMICs result in greater total antibiotic consumption (DDDs) in LMICs even though their consumption rate (and thus per capita use) is lower. In *B*, each bar reflects total consumption in the specified year for that country or group of countries. Data source: IQVIA MIDAS, 2000–2015, IQVIA Inc. All rights reserved (https://www.iqvia.com/solutions/commercialization/geographies/midas).

The increase in global consumption was primarily driven by increased consumption in LMICs. In 2000, HICs, led by France, New Zealand, Spain, Hong Kong, and the United States, had the highest antibiotic consumption rates. In 2015, four of the six countries with the highest consumption rates were LMICs (Turkey, Tunisia, Algeria, and Romania; Fig. 1 and *SI Appendix*, Fig. S1). In HICs, although the total amount of antibiotics consumed increased 6% between 2000 and 2015, from 9.7 to 10.3 billion DDDs, the antibiotic consumption rate decreased by a modest 4%, from 26.8 to 25.7 DDDs per 1,000 inhabitants per day (Fig. 2*A*). In LMICs, antibiotic consumption increased 114%, from 11.4 to 24.5 billion DDDs, and the antibiotic consumption rate increased 77%, from 7.6 to 13.5 DDDs per 1,000 inhabitants per day. Low- and lower-middle-income countries (LMICs-LM) accounted for a greater share of this increase than upper-middle-income countries (LMICs-UM): total antibiotic consumption in LMICs-LM increased 117%, from 8.1 to 17.5 billion DDDs, while, in LMICs-UM, antibiotic consumption increased 110%, from 3.3 to 6.9 billion DDDs (Fig. 2*B*). The antibiotic consumption rate in both LMICs-UM and LMICs-LM increased 78%, from 12.0 to 21.3 DDDs per 1,000 inhabitants per day, and from 6.7 to 11.9 DDDs per 1,000 inhabitants per day, respectively.

In 2015, the leading HIC consumers of antibiotics were the United States, France, and Italy, while the leading LMIC consumers were India, China, and Pakistan. Whereas antibiotic consumption in the three leading HICs marginally increased, the highest-consuming LMICs saw large increases. Between 2000 and 2015, antibiotic consumption increased from 3.2 to 6.5 billion DDDs (103%) in India, from 2.3 to 4.2 billion DDDs (79%) in China, and from 0.8 to 1.3 billion DDDs (65%) in Pakistan. The antibiotic consumption rate increased from 8.2 to 13.6 DDDs per 1,000 inhabitants per day (63%) in India, from 5.1 to 8.4 DDDs per 1,000 inhabitants per day (65%) in China, and from 16.2 to 19.6 DDDs per 1,000 inhabitants per day (21%) in Pakistan.

**Fig. 3.**
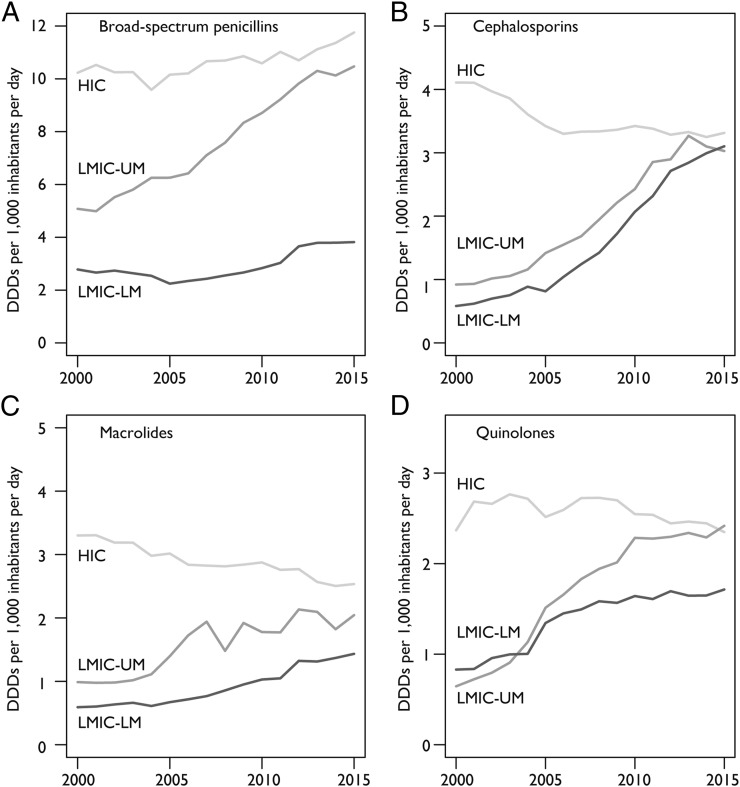
Antibiotic consumption rate for HICs, LMICs-UM, and LMICs-LM of the four most-consumed therapeutic classes of antibiotics in DDDs per 1,000 inhabitants per day. (*A*) Broad-spectrum penicillins, which correspond to the Anatomical Therapeutic Chemical (ATC) classification of penicillins with extended spectrum (J01CA) excluding carbenicillins. (*B*) Cephalosporins, which correspond to the ATC classification codes J01DB, J01DC, J01DD, and J01DE for the four generations of cephalosporins. (*C*) Macrolides, which correspond to the ATC classification for macrolides, lincosamides, and streptogramins (J01F). (*D*) Quinolones, which correspond to the ATC classification for quinolone antibacterials (J01M). Data source: IQVIA MIDAS, 2000–2015, IQVIA Inc. All rights reserved (https://www.iqvia.com/solutions/commercialization/geographies/midas).

The antibiotic consumption rate of broad-spectrum penicillins, the most commonly consumed class of antibiotics (39% of total DDDs in 2015), increased 36% between 2000 and 2015 globally. The greatest increase was in LMICs (56%), although the antibiotic consumption rate in HICs increased 15% (Fig. 3*A*). While the antibiotic consumption rate of the next three most consumed classes—cephalosporins (20% of total DDDs), quinolones (12% of total DDDs), and macrolides (12% of total DDDs)—all increased overall, the antibiotic consumption rate decreased in HICs. In LMICs, the antibiotic consumption rate increased 399, 125, and 119% for cephalosporins, quinolones, and macrolides, respectively, while the antibiotic consumption rate of these three drugs in HICs decreased by 18, 1, and 25%, respectively (Fig. 3 *B*–*D*).

**Fig. 4.**
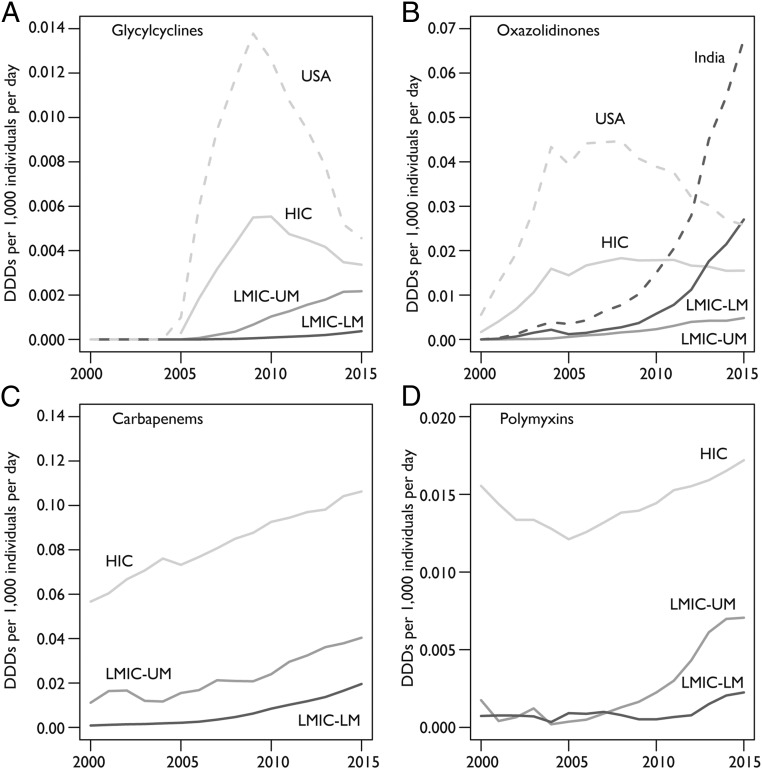
Antibiotic consumption rate for HICs, LMICs-UM, and LMICs-LM of new and last-resort antibiotics in DDDs per 1,000 inhabitants per day. (*A*) Glycylcyclines, which correspond to the ATC classification for tigecycline (J01AA12). (*B*) Oxazolidinones, which correspond to the ATC classifications for linezolid (J01XX08) and tedizolid (J01XX11). (*C*) Carbapenems, which correspond to the ATC classification for carbapenems (J01DH). (*D*) Polymyxins, which correspond to ATC classification for polymyxins (J01XB). Data source: IQVIA MIDAS, 2000–2015, IQVIA Inc. All rights reserved (https://www.iqvia.com/solutions/commercialization/geographies/midas).

Consumption of newer and last-resort antibiotic classes increased across all country income groups between 2000 and 2015. The United States was the largest consumer of glycylcyclines (tigecycline) and oxazolidinones (primarily linezolid as tedizolid was not introduced until 2014) through the late 2000s. However, the antibiotic consumption rate of these drugs in the United States began declining in 2009 (Fig. 4 *A* and *B*), and in 2015, Taiwan, Italy, Turkey, and Austria all had higher consumption rates for glycylcyclines than the United States, while India surpassed the United States antibiotic consumption rate for oxazolidinones in 2012 to become the highest consumer. The antibiotic consumption rate of carbapenems increased greatly in LMICs between 2000 and 2015 but remained far below consumption rates in HICs (Fig. 4*C*). Similarly, the antibiotic consumption rate of polymyxins (largely colistin) also increased in LMICs, particularly in LMICs-UM countries, but remained much lower than in HICs (Fig. 4*D*). The highest polymyxin consumption rates were in Spain, the United Kingdom, and Ireland, all of which had rates greater than 0.05 DDDs per 1,000 inhabitants per day in 2015.

We found a significant positive association between GDP per capita and changes in the antibiotic consumption rate in LMICs (*P* = 0.004), although no statistically significant association was found between these factors in HICs (*P* = 0.52). Other indicators, including the measles vaccination rate (which is a proxy for public health intervention capability), imports as a percentage of GDP, and physician density, were not correlated with changes in per capita antibiotic use across countries, irrespective of income group (Table 1).

**Table 1.** Fixed-effects regression analysis of factors associated with global antibiotic consumption (DDD per capita): 2000–2015

	Coefficient (SD)*	
Factor	Low- and middle-income countries	High-income countries
Log(GDP per capita)	3.14 (1.00)^†^	0.56 (0.70)
Percentage of children (12–23 mo) vaccinated for measles	0.04 (0.05)	0.07 (0.06)
Log(Imports as percentage of GDP)	−1.01 (1.01)	−0.20 (1.16)
Physician density per 1,000 population	1.39 (0.73)	0.49 (0.34)
Observations	302	305
Countries	39	32

Data source: IQVIA MIDAS, 2000–2015, IQVIA Inc. All rights reserved (https://www.iqvia.com/solutions/commercialization/geographies/midas).

* Both regressions are clustered by country to adjust for high serial correlation.

† *P* < 0.01.

**Fig. 5.**
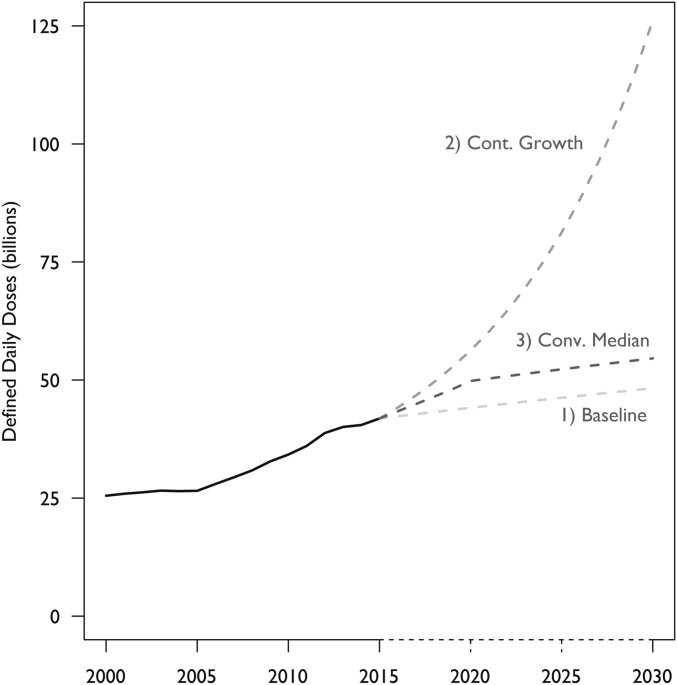
Projected total global antibiotic consumption (billions of DDDs): 2000–2030. Estimated global antibiotic consumption in all countries in billions of DDDs for three scenarios: (*i*) all countries continue to consume at current per capita rates; (*ii*) consumption of all countries continues to change at current compound annual growth rates; and (*iii*) all countries converge to the global median antibiotic consumption rate. Estimates were produced using antibiotic use data for 2000–2015 from the IQVIA MIDAS database and World Bank DataBank population estimates and projections for 2000–2030. Data source: IQVIA MIDAS, 2000–2015, IQVIA Inc. All rights reserved (https://www.iqvia.com/solutions/commercialization/geographies/midas).

Between 2000 and 2015, the estimated total global antibiotic consumption rate (including countries not reported in the IQVIA database) decreased slightly in HICs from 27.0 to 25.7 DDDs per 1,000 inhabitants per day in HICs and increased from 8.6 to 13.9 DDDs per 1,000 inhabitants per day in LMICs (*SI Appendix*, Fig. S2). Total global antibiotic consumption in 2015 was estimated to be 42.3 billion DDDs (15.8 DDDs per 1,000 inhabitants per day)—10.7 billion DDDs in HICs and 31.6 billion DDDs in LMICs. In our baseline condition, where we assumed no policy changes and constant antibiotic consumption rates set at current levels of use, global antibiotic use is projected to increase 15% between 2015 and 2030. If all countries continue to increase their antibiotic consumption rates at their compounded annual growth rates, we estimate that total consumption would increase 202% to 128 billion DDDs, while the antibiotic consumption rate would increase 161% to 41.1 DDDs per 1,000 inhabitants per day. Finally, if all countries converge on the global median antibiotic consumption rate in 2015 of 17.9 DDDs per 1,000 inhabitants per day by 2020, we estimate global antibiotic consumption would increase 32% to 55.6 billion DDDs (Fig. 5).

## Discussion

Using a global database of antibiotic sales, we found that antibiotic consumption rates increased dramatically in LMICs between 2000 and 2015, and in some LMICs have reached levels previously reported only in HICs. Overall consumption has also greatly increased, and the total amount of antibiotics consumed in LMICs, which was similar to HICs in 2000, was nearly 2.5 times that in HICs in 2015. Rising incomes are a major driver of increased antibiotic consumption in LMICs. Thus, although rates of antibiotic consumption in most LMICs remain below the general rate in HICs, barring major policy changes, they are expected to increase over time and converge, and possibly surpass, antibiotic consumption rates in HICs, in part due to the higher burden of infectious diseases in LMICs.

Tracking rates of antibiotic use is vitally important because of the well-quantified relationship between antibiotic use and resistance. However, although data on the burden of resistant bacterial infections is limited, both in HICs and especially in LMICs (2), the magnitude of the challenge posed by rising antibiotic resistance levels has become increasingly visible. Despite the emergence and spread of nearly untreatable infections, the global response to this public health crisis remains slow and inadequate. Reducing antibiotic consumption rates in HICs and slowing the growth rate of consumption in LMICs is urgently needed to contain the problem of resistance, particularly given the long timescales and resources necessary for development of new antibiotics. However, there is a need to balance access to essential medications, particularly in LMICs where the burden of infectious diseases likely still outweighs the burden of resistant infections and where in many countries there is a significant unmet need for antibiotics. Stewardship can improve judicious use without diminishing access to effective medications. Efforts to reduce unnecessary or inappropriate use based on awareness campaigns have resulted in lower antibiotic consumption rates in some high-consuming HICs (20). However, maintaining those efforts in the long run has proven challenging (21, 22), and the methods used in HICs may not be appropriate or feasible in LMICs. Research is urgently needed to understand the most effective methods for implementing stewardship programs in LMICs from the local to the national level in a manner that does not restrict antibiotics from those most burdened by treatable diseases.

Numerous studies have examined the drivers of consumption within and between HICs; however, the large variations in antibiotic consumption among countries are poorly explained. In our study, increases in the antibiotic consumption rate in LMICs were positively correlated with per capita GDP growth rates, but no similar relationship could be identified for HICs. Increases in economic growth provide access to goods and services, including antibiotics, which provides the most likely explanation for the positive relationship found between increasing wealth and increasing antibiotic consumption in LMICs. However, GDP growth in LMICs is also linked with increasing urbanization, which can facilitate the transmission of infectious diseases (23) and may contribute to the link between GDP growth and antibiotic consumption. Increasing incidence of bacterial infections, such as enteric fever (24), has been associated with rapid urbanization and increased consumption of antibiotics. Furthermore, rising incidence of nonbacterial infections associated with urbanization, such as dengue, chikungunya (25, 26), and viral diarrheal illnesses (27), is a significant driver of inappropriate consumption of antibiotics in LMICs. Additionally, declining air quality associated with urbanization and continued use of solid fuels for cooking also drives antibiotic consumption in LMICs by increasing the incidence of acute respiratory-tract infections (28).

The lack of a relationship between economic growth and antibiotic consumption in HICs suggests that access is not the leading factor driving differences between countries. Rather differences in consumption rates are driven by social and cultural norms regarding attitudes toward prescribing and use of antibiotics [see, e.g., Blommaert et al. (29)]. Therefore, embedding judicious use as a normative value in LMICs could prevent the future inappropriate use of antibiotics that currently plagues HICs. In addition, understanding the factors that result in lower antibiotic consumption rates in some HICs may help identify future policy solutions to promote similar trends in LMICs. However, additional studies in LMICs are needed to identify the regulatory and policy factors that result in lower consumption across these settings and whether they differ from higher-income settings.

We observed prominent differences in the rate of change in classes of antibiotics consumed in HICs and LMICs, particularly cephalosporins, consumption of which increased rapidly in LMICs while declining in HICs. These changes are concerning because the consumption of cephalsoporins, particularly third-generation cephalsoporins, is associated with emergence of extended spectrum beta-lactamase–producing bacteria (30). Consumption of other broad-spectrum agents like fluoroquinolones, macrolides, and second-line agents like oxazolidinones also increased in LMICs and decreased in HICs. In contrast, consumption of broad-spectrum penicillins, carbapenems, and polymyxins increased in both HICs and LMICs, although the rate of increase was faster in LMICs-UM than LMICs-LM. Changes in consumption patterns in LMICs likely reflect increasing access due to economic growth as noted above, as well as patent expiration, which reduces barriers to access as well as the cost of medications. However, the changing composition of consumption may also reflect alterations in patterns of resistance. For example, India, which had the greatest increase in antibiotic consumption in LMICs (65% between 2000 and 2015), had sustained economic growth (∼10% annual increase in GDP) through the 2000s. A large portion of this increase in consumption was due to an increase in the use of cephalosporins, which was likely due not only to economic growth, but also to changing prescribing practices for respiratory tract infections, skin and soft tissue infections, gonococcal infections, and enteric fever, where cephalosporins have replaced penicillins and quinolones for infection management due to rising resistance (31). Similarly, with increasing quinolone resistance in *Salmonella* Typhi (32), cephalosporins have become the treatment of choice for this infection in both outpatient (cefixime, cefpodoxime) and inpatient (ceftriaxone) settings (33). LMICs, with higher disease burdens, may be more sensitive to rising rates of resistance, which may drive local changes in antibiotic consumption patterns. However, a lack of surveillance for resistant infections makes it difficult to ascribe consumption changes to infectious disease burdens or resistance changes to overall consumption levels. Increased surveillance for resistance and infectious disease burden can improve the scientific basis for reducing antibiotic consumption.

Globally, the consumption of last-resort antibiotics—carbapenems and colistin—has been increasing. This rise is consistent with a well-documented increase in the number of infections resistant to carbapenems and colistin (34, 35). Given the recent finding that plasmid-mediated colistin resistance is spreading globally (35), this drug should be used prudently in humans and animals. Although consumption of the newer second-line drugs glycylcyclines and oxazolidinones declined in the United States after the Food and Drug Administration warned of a higher risk of death compared with other drugs used to treat similar infections (36, 37), globally the consumption of these drugs has increased. With rapidly growing incomes in LMICs, continued high disease burden, and increasing rates of resistance, an accelerated uptake of these and other new drugs can be expected compared with prior uptake rates for older drugs. This may lead to shorter time frames of effectiveness for new drugs, and as newer drugs are often less well-tolerated, an increase in adverse drug-related events.

While rising incomes provide increased access to medications, in resource-limited settings, financial barriers continue to restrict access to antibiotics particularly for the most vulnerable. This is further compounded by rising resistance to affordable first-line treatments, which already prevents the most vulnerable populations from accessing effective treatments in some countries (1). Innovative pricing mechanisms should be developed to improve access to lifesaving drugs for vulnerable populations (38) without compromising their future efficacy (31). Reducing the burden of disease is an alternative mechanism for reducing antibiotic consumption, particularly in countries where antibiotics are used as surrogates for other infection control measures (39). Investments in sanitation and improved hygiene measures were a major driving force in reducing the burden of infectious diseases in HICs in the 20th century. Large infrastructure projects in LMICs that improve the delivery of clean, safe water could reduce the burden of disease without increasing the use of antibiotics. For example, a recent study in India demonstrated that improved water quality could significantly reduce the burden of childhood diarrheal diseases, which in turn would reduce antibiotic consumption (40). Improved quality and quantity of water would likely improve hand hygiene compliance, particularly in healthcare settings. In HICs, numerous studies have found that improved hand hygiene helps reduce the consumption of antibiotics in the clinical setting (41). Access to vaccines and point-of-care diagnostics could similarly reduce unnecessary antibiotic consumption in resource-poor settings (42), both directly through vaccination against bacterial illnesses, such as the PCV (43, 44), or indirectly by reducing viral illnesses that often are treated unnecessarily with antibiotics. Policies to promote these alternatives to reduce consumption should be a major part of efforts to reduce antibiotic resistance. To aid policy development in this arena, future research efforts should quantify the economic benefit of investments by developed countries in these types of interventions relative to investments in the discovery of new antibiotic compounds.

To the best of our knowledge, IQVIA currently provides the only source of harmonized data on global antibiotic consumption. Without an alternative surveillance system to estimate global consumption, it is not possible to assess potential systematic bias in the database used for this study; however, our estimates are strongly correlated with data from the European Surveillance of Antimicrobial Consumption Network (ESAC-Net) (*SI Appendix*, Fig. S3), as well as others that have reported global antibiotic consumption (14, 16). While the DDD consumption data that we report permit our estimates to be directly compared with other sources of antibiotic consumption, including those from ESAC-Net, DDDs are not a perfect outcome measure of antibiotic prescribing, particularly for penicillins (14, 45). Indeed, for these drugs, the DDD is much lower than the actual prescribed dose, therefore overestimating antibiotic consumption. Also, the number of units per package and the amount of active substance per unit has increased over time in Europe and perhaps also in other continents (45). Despite these limitations, reporting of antibiotic consumption rates on a global scale is critical for carrying out effective policies to reduce consumption, such as setting and enforcing targets for antibiotic consumption based on current consumption levels (46) and inducing change by identifying the heaviest consumers of antibiotics (13). As part of the global action plan on AMR adopted by WHO, UN member states were urged to establish national action plans to combat antibiotic resistance, including reporting antibiotic consumption (11). However, additional study is needed to link antibiotic consumption rates of specific drugs and resistance rates of target pathogens to better inform policies to reduce antibiotic consumption.

With antibiotic consumption increasing worldwide, the challenge posed by antibiotic resistance is likely to get worse. As with climate change, there may be an unknown tipping point (47), and this could herald a future without effective antibiotics. Even in the absence of tipping points, the decline of antibiotic effectiveness represents a major threat to human health. Radical rethinking of policies to reduce consumption is necessary, including major investments in improved hygiene, sanitation, vaccination, and access to diagnostic tools both to prevent unnecessary antibiotic use and to decrease the burden of infectious disease. While more study is needed to understand the risks of radical reductions in consumption, immediate strategies are necessary to reduce mortality among the millions of people who die from resistant infections annually.

## Supplementary Material

Supplementary File
