# Supplementary material for: Global increase and geographic convergence in antibiotic consumption between 2000 and 2015
Source: Proc Natl Acad Sci U S A. 2018 Mar 26;115(15):E3463–70. doi: 10.1073/pnas.1717295115 (PMC5899442; doi:10.1073/pnas.1717295115)
Supplement: Supplementary File [file pnas.1717295115.sapp.pdf]

Supporting Information Appendix

for

Global increase and geographic convergence in antibiotic consumption between 2000 and 2015

Eili Y. Klein,[a,b,c] Thomas P. Van Boeckel,[d] Elena M. Martinez,[a] Suraj Pant,[a] Sumanth Gandra,[a] Simon A. Levin [e,f,g], Herman Goossens,[h] and Ramanan Laxminarayan[a,f,i]

[a] Center for Disease Dynamics, Economics & Policy, Washington, DC, USA

[b] Department of Emergency Medicine, Johns Hopkins School of Medicine, Baltimore, MD, USA

[c] Department of Epidemiology, Johns Hopkins Bloomberg School of Public Health, Baltimore, MD, USA

[d] Institute of Integrative Biology, Eidgenössische Technische Hochschule (ETH) Zürich, Switzerland

[e] Department of Ecology and Evolutionary Biology, Princeton University, Princeton, NJ, USA

[f] Princeton Environmental Institute, Princeton University, Princeton, NJ, USA

[g] Beijer Institute of Ecological Economics, Stockhom, Sweden

[h] Laboratory of Medical Microbiology, Vaccine & Infectious Diseases Institute, University of Antwerp, Antwerp, Belgium.

[i] Department of Global Health, University of Washington, Seattle, WA, USA

Supplementary Table 1. Years for which data were available by country/region and sector

	Sector			Sector	
**Country**	**Hospital**	**Retail**	**Country**	**Hospital**	**Retail**
Algeria	–	2002–2015	Lithuania†	2002–2015	2000–2015
Argentina	–	2000–2015	Luxembourg	–	2000–2015
Australia	2000–2015	2000–2015	Malaysia	2000–2015	2000–2015
Austria†	2005–2015	2000–2015	Mexico†	2005–2015	2000–2015
Bangladesh	–	2005–2015	Morocco	–	2000–2015
Belgium	2000–2015	2000–2015	Netherlands	2005–2015	2005–2015
Bosnia and Herzegovina	–	2011–2015	New Zealand	2000–2015	2000–2015
Brazil†	2005–2015	2000–2015	Norway	2000–2015	2000–2015
Bulgaria	2000–2015	2000–2015	Pakistan	–	2000–2015
Canada	2000–2015	2000–2015	Peru	–	2000–2015
Central America*	–	2000–2015	Philippines	2000–2015	2000–2015
Chile	–	2000–2015	Poland	2000–2015	2000–2015
China†	2000–2015	2011–2015	Portugal†	2005–2015	2000–2015
Colombia	–	2000–2015	Puerto Rico	2000–2015	2000–2015
Croatia	2005–2015	2005–2015	Romania†	2005–2015	2000–2015
Czech Republic	2000–2015	2000–2015	Russia	2000–2015	2000–2015
Denmark	–	2000–2015	Saudi Arabia	–	2000–2015
Dominican Republic	–	2000–2015	Serbia	–	2011–2015
Ecuador	–	2000–2015	Singapore	2000–2015	2000–2015
Egypt	–	2000–2015	Slovakia	2000–2015	2000–2015
Estonia	2002–2004	2000–2015	Slovenia	–	2000–2015
Finland	2000–2015	2000–2015	South Africa	2000–2015	2000–2015
France	2000–2015	2000–2015	South Korea	2000–2015	2000–2015
French West Africa**	–	2000–2015	Spain	2000–2015	2000–2015
Germany	2000–2015	2000–2015	Sri Lanka	–	2007–2015
Greece	–	2000–2015	Sweden	–	2000–2015
Hong Kong†	2000–2004	2000–2015	Switzerland	2000–2015	2000–2015
Hungary	2000–2015	2000–2015	Taiwan	2000–2015	2005–2015
India	2005–2015	2000–2015	Thailand	2000–2015	2000–2015
Indonesia†	2000–2004	2000–2015	Tunisia†	2011–2015	2000–2015
Ireland†	2006–2015	2000–2015	Turkey	2000–2015	2000–2015
Italy	2000–2015	2000–2015	Ukraine	2010–2015	2010–2015
Japan	2000–2015	2000–2015	United Arab Emirates	–	2000–2015
Jordan	–	2000–2015	United Kingdom	2000–2015	2000–2015
Kazakhstan†	2008–2015	2005–2015	United States	2000–2015	2000–2015
Kuwait	–	2000–2015	Uruguay	–	2000–2015
Latvia	–	2000–2015	Venezuela	–	2000–2015
Lebanon	–	2000–2015	Vietnam	2005–2015	2005–2015

Data Source: IQVIA MIDAS, 2000–2015, IQVIA Inc. All rights reserved. * Central America includes: Costa Rica, El Salvador, Guatemala, Honduras, Nicaragua, Panama; ** French West Africa includes: Benin, Cameroon, Burkina Faso, Chad, Cote d'Ivoire, Congo, Rep., Guinea, Mali, Niger, Senegal, Togo. † In these countries, hospital and retail data were both reported for some but not all years. To adjust for this, we used the ratio of antibiotic consumption in the hospital and retail sectors for the years both were reported to estimate the amount for the sector in the years not reported

Supplementary Methods

Identifying and estimating DDD unit values for conversion from SU to KG

Defined daily doses (DDDs) were calculated from kilogram data using the ATC/DDD index for 2016, developed by the WHO Collaborating Centre for Drug Statistics Methodology. DDD unit values were provided in the ATC/DDD index for 199 of the molecules in the IQVIA MIDAS database. When possible, DDD unit values not available through the ATC/DDD index were estimated from other sources (Supplementary Table 2). The DDD unit value for xibornol was identified through the MIMS Drug Information System, and the value for bacitracin was estimated using the conversion between international units and grams provided by etoolsage.com. For polymyxins, the sales data were not available in kilograms, so the largest-selling polymyxin product worldwide was chosen as the reference product to generate DDD values using information from Falagas and Kasiakou (1). Specifically, we assumed that 1 SU = 1 vial which was assumed to contain 80 mg of colistimethate sodium (1). As the ATC/DDD index has 3 million IU for the DDD, we converted to IU using the conversion rate of 12,500 IU per mg of colistimethate sodium. The same conversion was used for polymyxin B parenteral administration. For the remaining molecules for which no DDD unit value was available, DDD unit value was estimated as the average value for each antibiotic class by route of administration (Supplementary Table 3). In addition, several molecules had a route of administration for which no DDD unit value was available or were combination drugs for which no DDD unit value was specified, in these cases the DDD was calculated as the average DDD of other routes (Supplementary Table 4).

Supplementary Table 2: DDD Source for molecules with no DDD unit value in ATC/DDD database

Antibiotic	Route of administration	DDD (gm)	Source
Cefathiamidine	Parenteral	4	(2)
Cefroxadine	Oral	2.1	(3)
Cefteram Pivoxil	Oral	0.6	(3)
Faropenem	Oral	0.9	(3)
Lenampicillin	Oral	1	(3)
Lomefloxacin	Oral	0.6	(3)
Micronomicin	Parenteral	0.24	(4)
Polymyxin B	Oral	0.387	(3)
Tosufloxacin	Oral	0.6	(3)
Acetyl Kitasamycin	Oral	1.2	(4)
Astromicin	Parenteral	0.4	(3)
Bacitracin	Parenteral	0.68	See text
Balofloxacin	Oral	0.2	(4)
Ciclacillin	Oral	2	(3)
Garenoxacin	Oral	0.4	(3)
Gemifloxacin	Oral	0.32	(4)
Kitasamycin	Oral	1.2	(4)
Tebipenem	Oral	0.84	(3)
Xibornol	Oral/Parenteral	1	See text

Supplementary Table 3: DDDs for molecules with no DDD unit value in ATC/DDD database

Antibiotic	Class	Route of administration	DDD (gm)
Alatrofloxacin	Fluoroquinolones	Parenteral	0.5
Amoxicillin/Ampicillin	Broad-spectrum penicillins	Oral	1.5
Ampicillin/Sultamicillin	Broad-spectrum penicillins	Oral	1.5
Antofloxacin	Fluoroquinolones	Oral	0.5
Brodimoprim	Trimethoprim	Parenteral	0.4
Brodimoprim	Trimethoprim	Oral	0.6
Carfecillin	Carbenicillins	Oral	4.0
Cefacetrile	Cephalosporins	Oral	1.0
Cefoselis	Cephalosporins	Parenteral	3.3
Cephalosporin C	Cephalosporins	Parenteral	3.3
Etimicin	Aminoglycosides	Parenteral	0.5
Furbenicillin	Broad-spectrum penicillins	Oral	1.5
Kitasamycin	Macrolides	Parenteral	1.2
Meleumycin	Macrolides	Parenteral/Oral	1.3
Norvancomycin	Glycopeptides	Parenteral	1.3
Norvancomycin	Glycopeptides	Oral	2.0
Novobiocin	Glycopeptides	Parenteral	1.7

Novobiocin	Glycopeptides	Oral	2.0
Oritavancin	Glycopeptidess	Oral	1.2
Panipenem	Carbapenem	Oral	2.0
Penicillin G/Penicillin V	Narrow-spectrum penicillins	Oral	1.5
Polymyxin M	Polymyxins	Parenteral	0.2
Polymyxin M	Polymyxins	Oral	0.4
Roxithromycin	Macrolides	Parenteral	0.3
Spiramycin/Metronidazole	Macrolides	Parenteral	3.0
Sulfadiazine/Tetroxoprim	Trimethoprim	Parenteral/Oral	0.7
Sulfadiazine/Trimethoprim	Trimethoprim	Parenteral/Oral	1.0
Sulfadimidine/Trimethoprim	Trimethoprim	Parenteral	0.4
Sulfadimidine/Trimethoprim	Trimethoprim	Oral	1.0
Sulfadoxine/Trimethoprim	Trimethoprim	Parenteral	0.4
Sulfadoxine/Trimethoprim	Trimethoprim	Oral	0.6
Sulfalene/Trimethoprim	Trimethoprim	Parenteral	0.4
Sulfalene/Trimethoprim	Trimethoprim	Oral	0.6
Sulfamerazine/Trimethoprim	Trimethoprim	Parenteral	0.4
Sulfamerazine/Trimethoprim	Trimethoprim	Parenteral	0.8
Sulfamethizole/Trimethoprim	Trimethoprim	Oral	0.8
Sulfamethoxazole/Trimethoprim	Trimethoprim	Parenteral/Oral	1.9
Sulfametrole/Trimethoprim	Trimethoprim	Parenteral/Oral	1.9
Sulfamoxole/Trimethoprim	Trimethoprim	Oral	1.9
Tebipenem	Carbapenems	Parenteral	0.8
Telavancin	Glycopeptides	Parenteral	1.3
Telavancin	Glycopeptides	Oral	2.0

Supplementary Table 4: DDDs for molecules with a route of administration or a combination of molecules with no DDD unit value in ATC/DDD database

Antibiotic	Class	Route of administration	DDD (gm)
Amikacin	Aminoglycosides	Oral	1.0
Ampicillin/Clavulanic Acid	Broad-spectrum penicillins	Oral	6.0
Ampicillin/Sulbactam	Broad-spectrum penicillins	Oral	6.0
Arbekacin	Aminoglycosides	Oral	0.2
Astromicin	Aminoglycosides	Oral	0.4
Azidocillin	Narrow-spectrum penicillins	Parenteral	1.5
Aztreonam	Monobactams	Oral	4.0
Bekanamycin	Aminoglycosides	Oral	0.6
Biapenem	Carbapenems	Oral	1.2
Carbenicillin	Carbenicillins	Oral	12.0
Carindacillin	Carbenicillins	Parenteral	4.0
Carumonam	Monobactams	Oral	2.0
Cefadroxil	Cephalosporins	Parenteral	2.0
Cefalexin	Cephalosporins	Parenteral	2.0

Cefazolin	Cephalosporins	Oral	3.0
Cefdinir	Cephalosporins	Parenteral	0.6
Cefditoren Pivoxil	Cephalosporins	Parenteral	0.4
Cefepime	Cephalosporins	Oral	2.0
Cefixime	Cephalosporins	Parenteral	0.4
Cefotaxime	Cephalosporins	Oral	4.0
Cefotetan	Cephalosporins	Oral	4.0
Cefpirome	Cephalosporins	Oral	4.0
Cefpodoxime Proxetil	Cephalosporins	Parenteral	0.4
Ceftazidime	Cephalosporins	Oral	4.0
Ceftizoxime	Cephalosporins	Oral	4.0
Ceftriaxone	Cephalosporins	Oral	2.0
Chlortetracycline	Tetracyclines	Parenteral	1.0
Clofoctol	Other	Parenteral/Oral	1.5
Clometocillin	Narrow-spectrum penicillins	Parenteral	1.0
Colistin	Polymyxins	Oral	0.2
Dalbavancin	Glycopeptides	Oral	1.5
Dalfopristin/Quinupristin	Macrolides	Oral	1.5
Daptomycin	Lipopeptides	Oral	0.3
Demeclocycline	Tetracyclines	Parenteral	0.6
Dibekacin	Aminoglycosides	Oral	0.1
Dirithromycin	Macrolides	Parenteral	0.5
Doripenem	Carbapenems	Oral	1.5
Enoxacin	Fluoroquinolones	Parenteral	0.8
Ertapenem	Carbapenems	Oral	1.0
Erythromycin Stinoprate	Macrolides	Parenteral	1.0
Flurithromycin	Macrolides	Parenteral	0.8
Gentamicin	Aminoglycosides	Oral	0.2
Imipenem/Cilastatin	Carbapenems	Oral	2.0
Josamycin	Macrolides	Parenteral	2.0
Kanamycin	Aminoglycosides	Oral	1.0
Lomefloxacin	Fluoroquinolones	Parenteral	0.6
Mecillinam	Broad-spectrum penicillins	Oral	1.2
Meropenem	Carbapenems	Oral	2.0
Metronidazole	Other	Oral	0.9
Micronomicin	Aminoglycosides	Oral	0.2
Midecamycin	Macrolides	Oral	1.0
Norfloxacin	Fluoroquinolones	Parenteral	0.8
Oleandomycin	Macrolides	Parenteral	1.0
Paromomycin	Aminoglycosides	Parenteral	3.0
Penamecillin	Narrow-spectrum penicillins	Parenteral	1.0
Penicillin G	Narrow-spectrum penicillins	Oral	3.6
Penicillin G/Streptomycin	Streptomycin combinations	Oral	1.0
Penicillin G/Penicillin V/Streptomycin	Streptomycin combinations	Oral	1.0

Penicillin V	Narrow-spectrum penicillins	Parenteral	2.0
Pheneticillin	Narrow-spectrum penicillins	Parenteral	2.0
Pivmecillinam	Broad-spectrum penicillins	Oral	1.4
Pivmecillinam	Broad-spectrum penicillins	Parenteral	0.6
Pristinamycin	Macrolides	Parenteral	2.0
Ribostamycin	Aminoglycosides	Oral	1.0
Rokitamycin	Macrolides	Parenteral	0.8
Rolitetracycline	Tetracyclines	Oral	0.3
Sisomicin	Aminoglycosides	Parenteral	0.3
Teicoplanin	Glycopeptides	Oral	0.4
Telavancin	Glycopeptides	Oral	0.4
Ticarcillin	Carbenicillins	Oral	15.0
Tigecycline	Glycylcycline	Oral	0.1
Tobramycin	Aminoglycosides	Oral	0.2

Estimating total global antibiotic use in 2000–2015

Total global antibiotic use was predicted in DDDs. Population data for countries not included in the MIDAS database were retrieved from the World Development Indicators in the World Bank DataBank. Average per capita antibiotic consumption was calculated for each year and national income group. Total antibiotic use in countries not included in the IQVIA MIDAS database was estimated by multiplying the country's population in each year by this average. This method was also used to predict antibiotic use in countries included in the IQVIA MIDAS database that were missing antibiotic use data in all sectors for some years.

Predicting total global antibiotic use in 2015–2030

Total global antibiotic use in DDDs between 2015 and 2030 was predicted using three scenarios of future antibiotic use. Population projections were retrieved from the World Bank DataBank for all countries except Taiwan. Population projections for Taiwan were retrieved from the Taiwan National Development Council. The World Bank data were missing population

projections for some countries; future populations for these countries were predicted using the average population growth rate in the last five years of available data.

In scenario 1, all countries continue consuming at their per capita rate of antibiotic use in 2015, accounting for changes in population. In scenario 2, all countries' per capita antibiotic consumption continues increasing at current growth rates. Current growth rate was defined as the compound annual growth rate in per capita antibiotic use in each country between 2010 and 2015. In scenario 3, all countries converged to the 2015 global median per capita antibiotic use rate by 2020, and then continued to consume at this global median through 2030 (Figure 5).

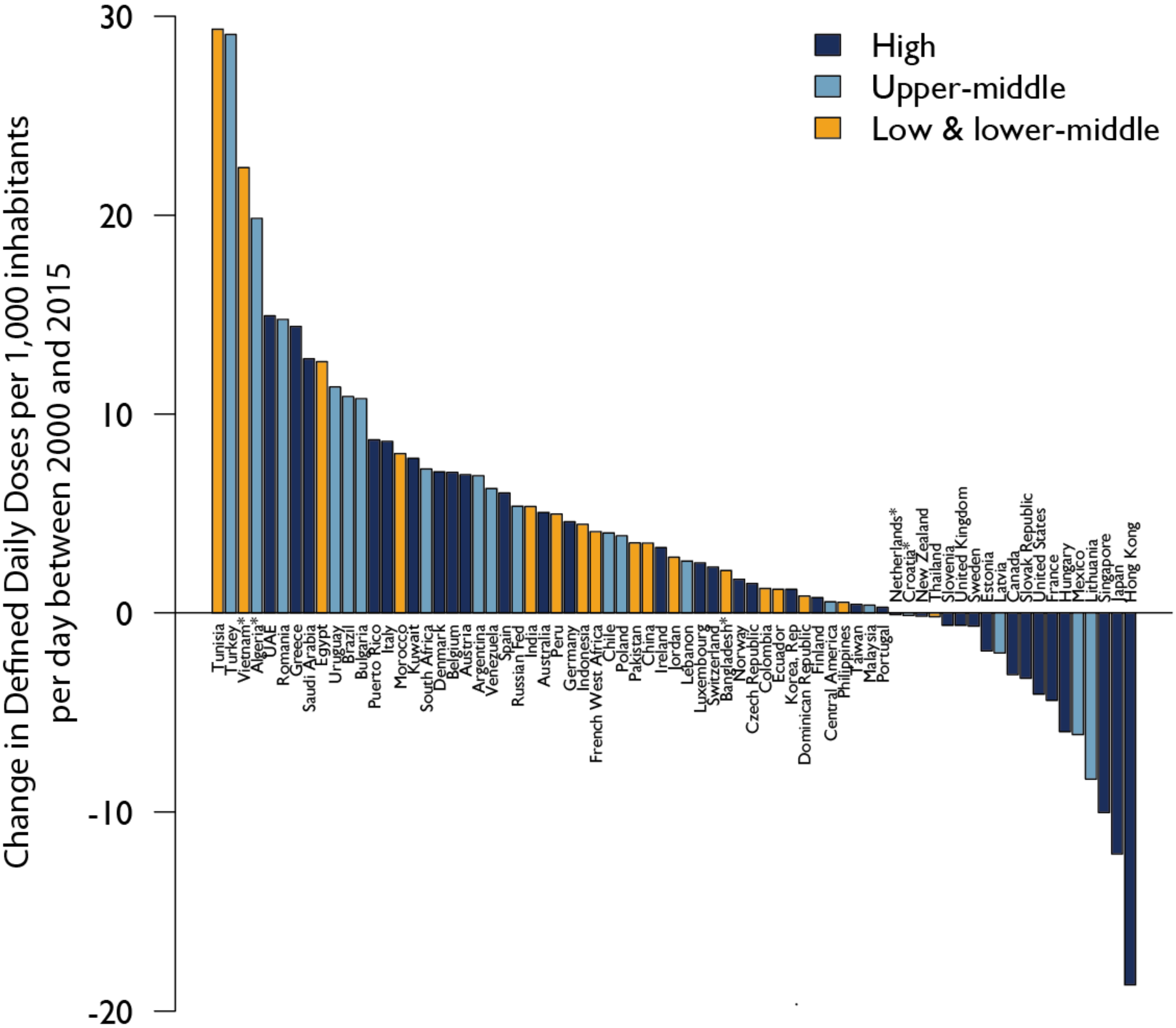

Supplementary Figure 1. Change in the national antibiotic consumption rate between 2000 and 2015 in defined daily doses per 1,000 inhabitants per day (DDDs per 1,000 inhabitants per day). For Vietnam, Bangladesh, Netherlands, and Croatia change was calculated from 2005 as data prior to that year were not available. For Algeria, change was calculated from 2002 as data prior to that year were not available. Data source: IQVIA MIDAS, 2000–2015, IQVIA Inc. All rights reserved.

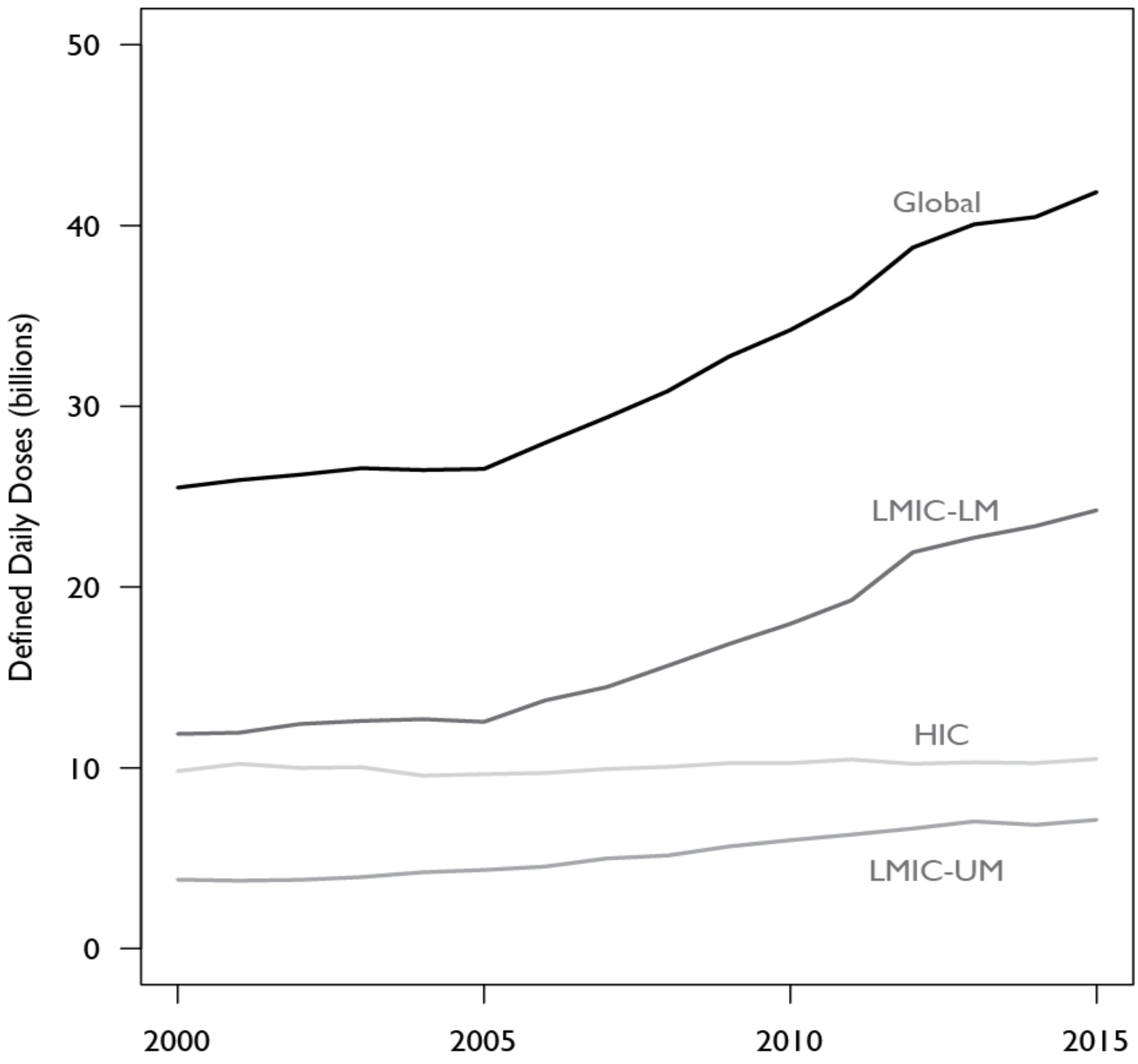

Supplementary Figure 2. Estimated total global antibiotic consumption, 2000–2015, by income group: High-income countries (HIC), upper-middle-income countries (LMIC-UM), and low- & lower-middle-income countries (LMIC-LM). Antibiotic consumption is estimate of total consumption for all countries in billions of DDDs, including extrapolations to countries not included in QuantilesIMS MIDAS database, broken down by country income group. Totals were estimated using antibiotic use data from 2000–2015 from the IQVIA MIDAS database and World Bank DataBank population estimates for 2000–2015. Extrapolated data was estimated by income group (see supplementary methods). Data source: World Bank DataBank and IQVIA MIDAS, 2000–2015, IQVIA Inc. All rights reserved.

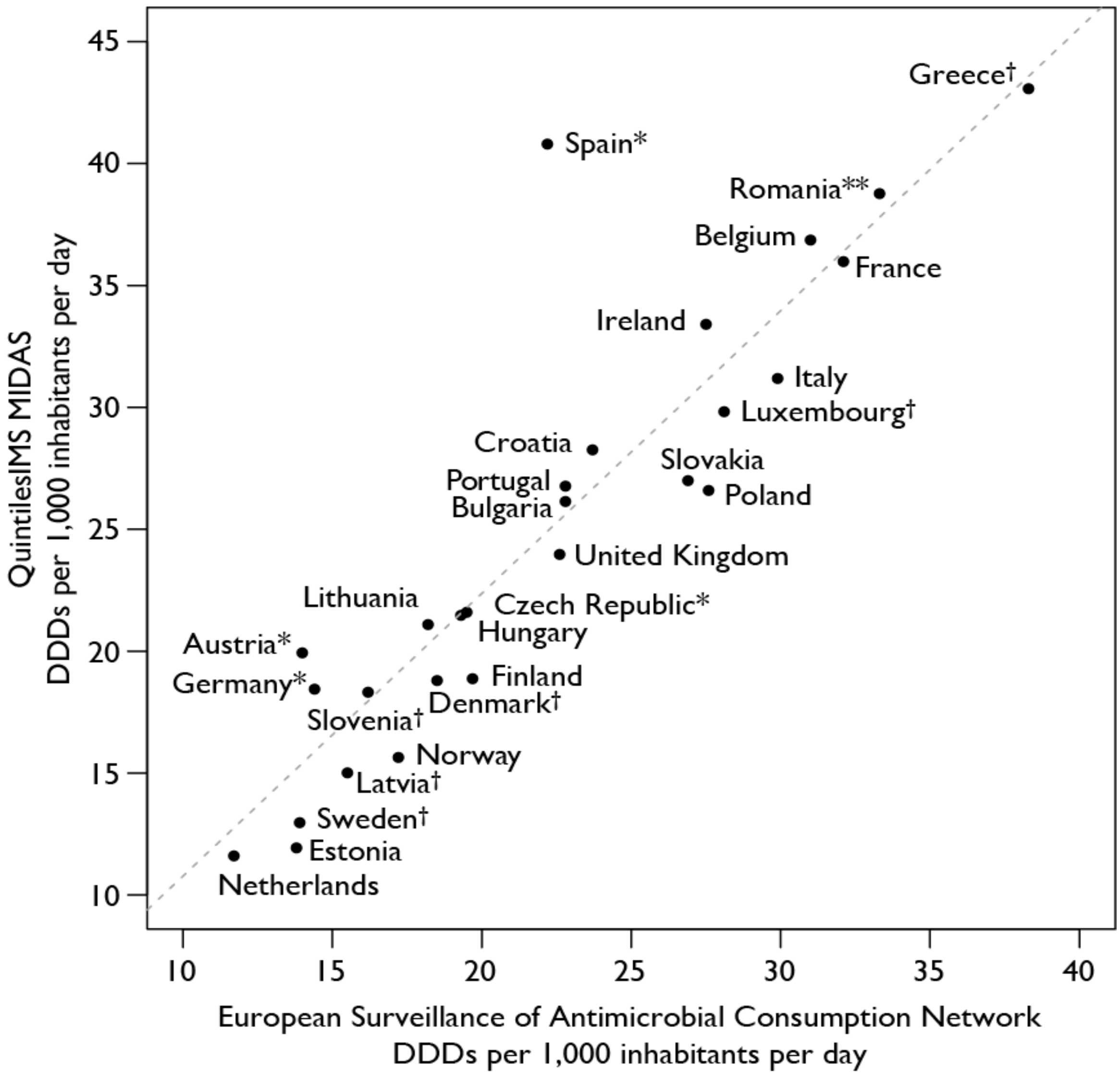

Supplementary Figure 3. Correlation between antibiotic consumption rates from the European Centre for Disease Prevention and Control's European Surveillance of Antimicrobial Consumption Network (ESAC-Net) and the IQVIA MIDAS database. Each point represents the defined daily doses (DDDs) per 1,000 inhabitants per day in 2015. The gray dashed line is the linear regression of the two sets of values. The correlation coefficient between the two sets of values was 93%. * Countries provided only community (primary care) data but not hospital data to ESAC-Net. ** Countries provided only total care data to ESAC-Net. †Data was only available from IQVIA for retail sector. Data source: World Bank DataBank, ESAC-Net and IQVIA MIDAS, 2000–2015, IQVIA Inc. All rights reserved.

References

1. Falagas ME, Kasiakou SK (2006) Use of international units when dosing colistin will help decrease confusion related to various formulations of the drug around the world. *Antimicrob Agents Chemother* 50(6):2274–2275.

2. Zhang W, et al. (2008) Antibiotic use in five children's hospitals during 2002–2006: the impact of antibiotic guidelines issued by the Chinese Ministry of Health. *Pharmacoepidemiol Drug Saf* 17(3):306–311.

3. Muraki Y, et al. (2016) Japanese antimicrobial consumption surveillance: First report on oral and parenteral antimicrobial consumption in Japan (2009–2013). *J Glob Antimicrob Resist* 7:19–23.

4. Dongsook Kim, Namsoon Kim, Sukhyang Lee (2007) Determination of Defined Daily Dose of Medicines using Nominal Group Technique and Analysis of Antibiotics Use in National Insurance Claim Data: Focused on Antibiotics without DDD of WHO. *Kor J Clin Pharm* 17(1):19–32.
